# Breaking of Henry’s law for sulfide liquid–basaltic melt partitioning of Pt and Pd

**DOI:** 10.1038/s41467-021-26311-x

**Published:** 2021-10-13

**Authors:** Mingdong Zhang, Yuan Li

**Affiliations:** 1 State Key Laboratory of Isotope Geochemistry, Guangzhou Institute of Geochemistry, Chinese Academy of Sciences, 510640 Guangzhou, China; 2 CAS Center for Excellence in Deep Earth Science, 510640 Guangzhou, China; 3 College of Earth and Planetary Sciences, University of the Chinese Academy of Sciences, 100049 Beijing, China

**Keywords:** Planetary science, Geochemistry

## Abstract

Platinum group elements are invaluable tracers for planetary accretion and differentiation and the formation of PGE sulfide deposits. Previous laboratory determinations of the sulfide liquid–basaltic melt partition coefficients of PGE (${D}_{PGE}^{SL/SM}$) yielded values of 10^2^–10^9^, and values of >10^5^ have been accepted by the geochemical and cosmochemical society. Here we perform measurements of ${D}_{Pt,\,Pd}^{SL/SM}$ at 1 GPa and 1,400 °C, and find that ${D}_{Pt,\,Pd}^{SL/SM}$ increase respectively from 3,500 to 3.5 × 10^5^ and 1,800 to 7 × 10^5^, as the Pt and Pd concentration in the sulfide liquid increases from 60 to 21,000 ppm and 26 to 7,000 ppm, respectively, implying non-Henrian behavior of the Pt and Pd partitioning. The use of ${D}_{Pt,\,Pd}^{SL/SM}$ values of 2,000–6,000 well explains the Pt and Pd systematics of Earth’s mantle peridotites and mid-ocean ridge basalts. Our findings suggest that the behavior of PGE needs to be reevaluated when using them to trace planetary magmatic processes.

## Abstract (web summary)

Platinum group elements are used as tracers for planetary and PGE sulfide deposit formation. Here, the authors, through the measurements of Pt and Pd partition coefficients between sulfide liquid and basaltic melt, demonstrate that the partitioning of Pt and Pd does not obey Henry’s law.

## Introduction

Platinum group elements (PGE: Os, Ir, Ru, Rh, Pt, and Pd) are powerful tracers for planetary accretion and core–mantle–crust differentiation^1–5^, as well as the formation of magmatic PGE sulfide deposits in Earth’s crust^4,6,7^. In planetary magmatic systems, PGE are primarily controlled by their partitioning into sulfide liquid. However, experimental determinations on the partition coefficients of PGE between sulfide liquid and silicate (basaltic) melt (${D}_{PGE}^{SL/SM}$) have yielded widely discrepant results, with ${D}_{PGE}^{SL/SM}$ values ranging from 10^2^ to 10^9^ (refs. ^4,8–13^). Studies in the 1990s determined ${D}_{PGE}^{SL/SM}$ by performing experiments at 1 bar^10–12^. Using a bulk analytical technique, i.e., neutron activation analysis, the obtained ${D}_{PGE}^{SL/SM}$ for a given element usually vary in the order of 10^2^–10^6^, which was late ascribed to inefficient separation of sulfide and silicate phases and/or the presence of PGE-sulfide nuggets in the silicate melt^8^. Experimental studies in the 2000s determined ${D}_{PGE}^{SL/SM}$ by measuring the solubility of PGE in sulfide liquid and silicate melt separately, which usually yielded ${D}_{PGE}^{SL/SM}$ values in the order of 10^5^–10^9^ (refs. ^14–16^). However, the disadvantage of this “indirect” method is that the formation of PGE-sulfide species in the silicate melt was not considered, which may result in artificially high ${D}_{PGE}^{SL/SM}$ (refs. ^4,8,17^). Most recent experimental studies determined ${D}_{PGE}^{SL/SM}$ using the in-situ micro-analytical technique of laser ablation–inductively coupled plasma–mass spectroscopy (LA–ICP–MS), such that the contribution of PGE-sulfide nuggets in the silicate melt can be identified and filtered^4,18^. The most precise ${D}_{PGE}^{SL/SM}$ values obtained at 1200 °C and 1 bar by Mungall and Brenan are larger than 10^5^ (ref. ^4^), which have been widely accepted and used by the geochemical and cosmochemical society since their publication in 2014 (refs. ^7,8,19,20^). However, in ref. ^4^, a few wt.% of PGE was doped in the sulfide liquid to facilitate the analysis of PGE in the silicate melt. Whether ${D}_{PGE}^{SL/SM}$ obey Henry’s law when the sulfide liquid has such high PGE concentration has never been investigated. Using the bulk analytical technique, M.E Fleet, J.H Crocket and coauthors first recognized the dependence of ${D}_{PGE}^{SL/SM}$ on the PGE concentration in the sulfide liquid in the 1990s (refs. ^11,12^). However, this has not been further investigated since then, although LA–ICP–MS has been one of the most powerful in-situ micro-analytical techniques from the 2000s. Here we present experimental measurements of ${D}_{Pt,\,Pd}^{SL/SM}$ as a function of the Pt and Pd concentration in the sulfide liquid (${C}_{Pt,\,Pd}^{SL}$) using the in-situ analytical technique of LA–ICP–MS. We find that ${D}_{Pt,\,Pd}^{SL/SM}$ increase with increasing ${C}_{Pt,\,Pd}^{SL}$, and the use of ${D}_{Pt,\,Pd}^{SL/SM}$ values of < 10^4^, which correspond to the ${C}_{Pt,\,Pd}^{SL}$ in Earth’s magmatic systems, can well explain the observed Pt and Pd systematics of Earth’s mantle peridotites and mid-ocean ridge basalts (MORB). These findings suggest that non-Henrian partitioning behavior of PGE should be considered when applying laboratory-determined ${D}_{PGE}^{SL/SM}\,$in planetary magmatic systems.

## Results and discussion

### Pt and Pd partitioning between sulfide liquid and basaltic melt

**Fig. 1:**
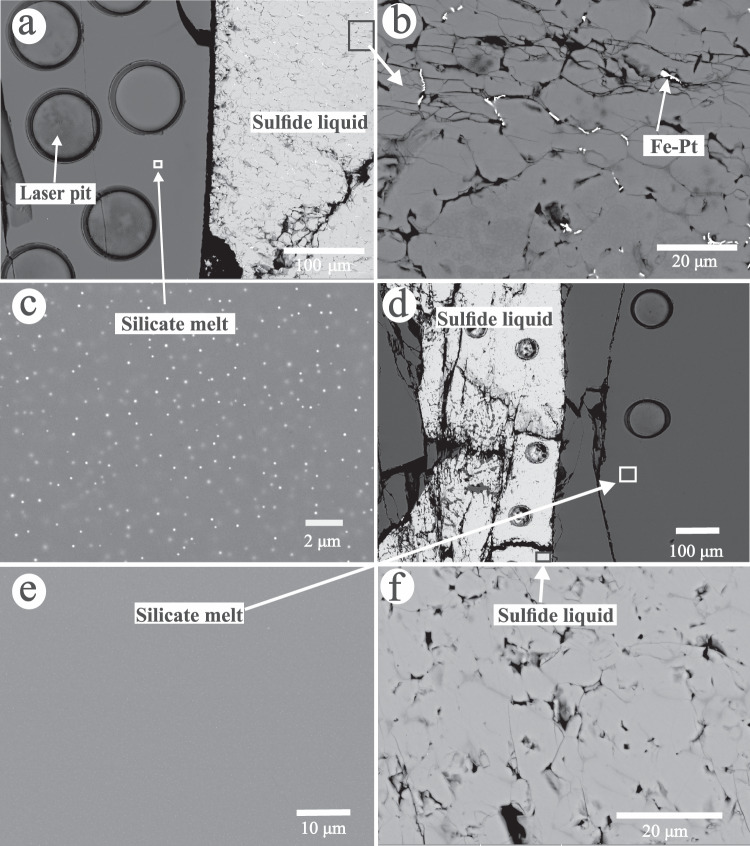
Selected back-scattered electron images showing characteristic features of the run products of sulfide liquid–basaltic melt partitioning experiments. **a** BSE image of run Z14, showing coexisting sulfide liquid and silicate melt in a graphite capsule. **b** Closer view of the quenched sulfide liquid with 5300 ppm Pt from run Z14, which includes large pyrrhotite crystals and exsolved Fe-Pt alloys at the boundary of pyrrhotite crystals. **c** Closer view of the quenched silicate melt in (**a**), showing homogeneously distributed small sulfide dots (< 0.1 µm) produced during quench. d BSE image of one reversal experiment (run Z28) for Pd partitioning, showing coexisting sulfide liquid and silicate melt. **e** Closer view of the quenched silicate melt in (**d**). **f** Closer view of the quenched sulfide liquid with 163 ppm Pd in (**d**).

Two sets of experiments were performed at 1 GPa and 1400 °C using a piston cylinder apparatus (Supplementary Data 1 and Methods). In Set-1 of 18 forward experiments, ~60–70 wt.% basalt, and ~30–40 wt.% sulfide (FeS) doped with 60–21,000 ppm Pt or 30–7000 ppm Pd, were loaded in graphite capsules. In Set-2 of four reversal experiments, ~200–7000 ppm Pd or 1000 ppm Pt was physically mixed with the basalt and then, ~60–70 wt.% basalt together with ~30–40 wt.% sulfide was loaded in graphite capsules. The formation of PGE nuggets in the silicate melt is related to the oxidation state of the starting silicate^21^. In order to reduce the formation of Pt or Pd nuggets in our basaltic melt, the starting basalt was prepared to contain only FeO without Fe_2_O_3_. The experimental durations ranged from 24 to 96 h. The experimental oxygen fugacity was close to the C–CO_2_ buffer^22–24^. All experiments produced coexisting basaltic melt and sulfide liquid. The basaltic melt was quenched into glass, and the sulfide liquid was qunched into large pyrrhotite crystals and minor Fe-Pt or Fe-Pd alloys, as shown in Fig. 1 and Supplementary Fig. 1. In the quenched sulfide liquid, the proportion of the Fe-Pt or Fe-Pd alloys increases with the mass of Pt or Pd doped in the experiments, and they occur alone the bouduary of pyrrhotite crystals. We interpret the Fe-Pt or Fe-Pd alloys as resulting from exsolution of sulfide liquid during quench, as interpreted previouly^4^. We found very small sulfide dots (< 0.1 µm) homogeneously distributed in the basaltic glass from all experiments, which we interpret as resulting from quench because of the significant drop in sulfide solubility (Fig. 1 and Supplementary Fig. 1). In a few experiments, we also found that Pt- or Pd-sulfide droplets (1–5 µm) dispersed as nuggets/inclusions in part of the basaltic glass, which we interpret as resulting from ineffective segregation from the silicate melt during the run (Supplementary Fig. 1e, f). The basaltic glasses that do not contain macroscopically visible sulfide nuggets (1–5 µm) are our analytical targets.

**Fig. 2:**
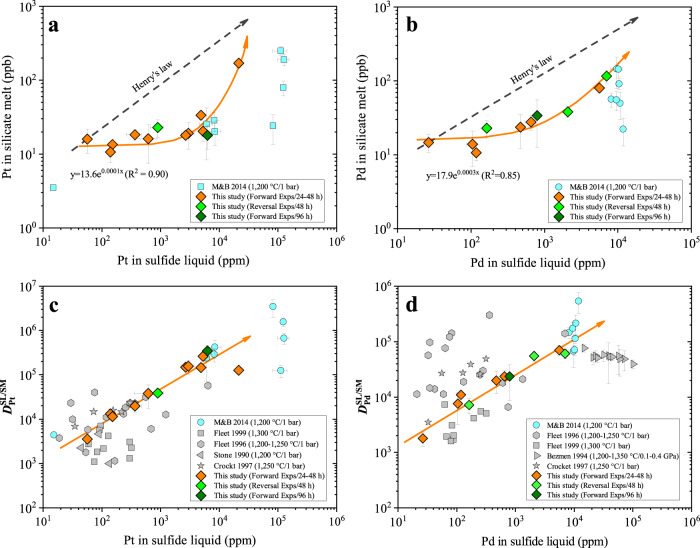
Dependence of the Pt and Pd concentration in the silicate melt and ${D}_{Pt,\,Pd}^{SL/SM}$ on the Pt and Pd concentration in the sulfide liquid. **a**, **b** The Pt and Pd concentration in the silicate melt increases exponentially with increasing the Pt and Pd concentration in the sulfide liquid. The dashed gray lines show the co-variation of the Pt and Pd concentration in the silicate melt and sulfide liquid if the partitioning of Pt and Pd between sulfide liquid and silicate melt obeys Henry’s law. **c**, **d** ${D}_{Pt,\,Pd}^{SL/SM}$ increase linearly with increasing the Pt and Pd concentration in the sulfide liquid. The literature data are taken from M&B 2014 (ref. ^4^), Fleet 1996 (ref. ^10^), Fleet 1999 (ref. ^12^), Stone 1990 (ref. ^9^), Bezmen 1994 (ref. ^13^), and Crocket 1997 (ref. ^11^). Note that the literature data of M&B 2014, obtained using in-situ micro-analytical technique of LA–ICP–MS, are systematically consistent with the present results. The other literature data (filled gray symbols) were obtained by neutron activation analysis of bulk samples, and an effective separation of the silicate and sulfide phases was questioned^8^. Source data are provided in Supplementary Data 1.

Major and trace element compositions of the quenched basaltic melt and sulfide liquid were measured using electron probe microanalyzer (EPMA) and LA–ICP–MS with large beam sizes (Methods and Supplementary Data 2, 3). The time-resolved LA–ICP–MS signals of Pt and Pd are constant for most of the analyses, confirming the absence of Pt- or Pd-sulfide nuggets in the analyzed basaltic melt from both forward and reversal experiments (Supplementary Fig. 2). Any LA–ICP–MS analyses with Pt or Pd signals contaminated by sulfide nuggets in the silicate melt, as shown in Supplementary Fig. 2g, were discarded. The analytical results show that ${C}_{Pt,\,Pd}^{SL}$ equals 60–21,000 ppm and 26–7000 ppm (parts per million by weight), respectively, and the ${C}_{Pt,\,Pd}^{SL}$ values obtained using EPMA and LA–ICP–MS agree within 20% relative (Supplementary Fig. 3). ${C}_{Pt,\,Pd}^{SM}$ equals 10–160 ppb and 10–120 ppb (parts per billion by weight), respectively, which increases exponentially with increasing ${C}_{Pt,\,Pd}^{SL}$ (Fig. 2a, b and Supplementary Data 1). Nernst partition coefficients ${D}_{Pt,\,Pd}^{SL/SM}$, as calculated from the weight proportions of Pt and Pd in the sulfide liquid (${C}_{Pt,\,Pd}^{SL}$) and basaltic melt (${C}_{Pt,\,Pd}^{SM}$), range from 1800 to 7 × 10^5^, which increase linearly with increasing the Pt and Pd concentration in the sulfide liquid (Fig. 2c, d and Supplementary Data 1). No correlation was observed between ${D}_{Pt,\,Pd}^{SL/SM}$, the FeO content in the silicate melt, or the atomic metal/S ratio in the sulfide liquid (Supplementary Figs. 4 and 5). At a given Pt and Pd concentration in the sulfide liquid, ${C}_{Pt,\,Pd}^{SL}$ and ${D}_{Pt,\,Pd}^{SL/SM}$ do not vary as a function of the experimental duration, and the forward and reversal experiments yielded consistent ${C}_{Pt,\,Pd}^{SL}$ and ${D}_{Pt,\,Pd}^{SL/SM}$ (Fig. 2). These observations suggest the approach of equilibrium partitioning of Pt and Pd. Previous ${C}_{Pt,\,Pd}^{SM}$ and ${D}_{Pt,\,Pd}^{SL/SM}$, as obtained by Mungall and Brenan using LA–ICP–MS (ref. ^4^), are systematically consistent with the present results (Fig. 2).

### Non-Henrian behavior of ${D}_{Pt,\,Pd}^{SL/SM}$

Both the non-linear increase of ${C}_{Pt,\,Pd}^{SM}$ and the linear increase of ${D}_{Pt,\,Pd}^{SL/SM}$ with increasing ${C}_{Pt,\,Pd}^{SL}$ (Fig. 2) indicate that the partitioning of Pt and Pd between sulfide liquid and basaltic melt follows a non-Henrian behavior. Assuming a chalcophile element M dissolves as M-oxide in the silicate melt, the partitioning of M between sulfide liquid and silicate melt can be described by the following exchange reaction:

1 $${{{{{{\rm{MO}}}}}}}_{n/2}({{{{{\rm{SM}}}}}})+n/2{{{{{\rm{FeS}}}}}}\,({{{{{\rm{SL}}}}}})\,={{{{{{\rm{MS}}}}}}}_{n/2}({{{{{\rm{SL}}}}}})+n/2{{{{{\rm{FeO}}}}}}({{{{{\rm{SM}}}}}})$$

where *n* denotes the valence state of M. The equilibrium constant (*k*) of Eq. (1) can be written as:

2 $${k}_{{{{{{\rm{Eq}}}}}}.(1)}=\frac{{\alpha }_{M{S}_{n/2}}^{SL}\cdot {({\alpha }_{FeO}^{SM})}^{n/2}}{{\alpha }_{M{O}_{n/2}}^{SM}\cdot {({\alpha }_{FeS}^{SL})}^{n/2}}$$

and

3 $${k}_{{{{{{\rm{Eq}}}}}}.(1)}=\frac{{x}_{M{S}_{n/2}}^{SL\,}\cdot {\gamma }_{M{S}_{n/2}}^{SL\,}\cdot {({x}_{FeO}^{SM})}^{n/2}\cdot {({\gamma }_{FeO}^{SM})}^{n/2}}{{x}_{M{O}_{n/2}}^{SM\,}\cdot {\gamma }_{M{O}_{n/2}}^{SM\,}\cdot {({x}_{FeS}^{SL})}^{n/2}\cdot {({\gamma }_{FeS}^{SL})}^{n/2}}$$

In Eqs. (2) and (3) $\alpha$, $x$, and $\gamma$ denote activity, mole fraction, and activity coefficient, respectively, such as ${\alpha }_{M{S}_{n/2}}^{SL}$ denoting the activity of $M{S}_{n/2}$ in the sulfide liquid. Rearranging Eq. (3) would yield:

4 $$\frac{{x}_{M{S}_{n/2}}^{SL\,}}{{x}_{M{O}_{n/2}}^{SM\,}}={k}_{{{{{{\rm{Eq}}}}}}.(1)}\cdot \frac{{\gamma }_{M{O}_{n/2}}^{SM\,}\cdot {({x}_{FeS}^{SL})}^{n/2}\cdot {({\gamma }_{FeS}^{SL})}^{n/2}}{\,{\gamma }_{M{S}_{n/2}}^{SL\,}\cdot {({x}_{FeO}^{SM})}^{n/2}\cdot {({\gamma }_{FeO}^{SM})}^{n/2}\,}$$

Since ${D}_{M}^{SL/SM}$ equals $C\cdot \frac{{x}_{M{S}_{n/2}}^{SL\,}}{{x}_{M{O}_{n/2}}^{SM\,}}$, where $C$ is a constant, the following equation can be obtained:

5 $${D}_{M}^{SL/SM}={k}_{{{{{{\rm{Eq}}}}}}.(1)}\cdot \frac{{\gamma }_{M{O}_{n/2}}^{SM\,}\cdot {({x}_{FeS}^{SL})}^{n/2}\cdot {({\gamma }_{FeS}^{SL})}^{n/2}}{\,{\gamma }_{M{S}_{n/2}}^{SL\,}\cdot {({x}_{FeO}^{SM})}^{n/2}\cdot {({\gamma }_{FeO}^{SM})}^{n/2}\,}\cdot \frac{1}{C}$$

All our experiments were performed at 1 GPa and 1400 °C with very similar silicate melt compositions (Supplementary Data 2); therefore, both ${k}_{{{{{{\rm{Eq}}}}}}.(1)}$ and ${({x}_{FeO}^{SM})}^{n/2}\cdot {({\gamma }_{FeO}^{SM})}^{n/2}$ are constant in our experiments. The sulfide liquid contains 60–21,000 ppm Pt or 26–7000 ppm Pd. We therefore assume that ${({x}_{FeS}^{SL})}^{n/2}\cdot {({\gamma }_{FeS}^{SL})}^{n/2}$ is also constant in our experiments. Accordingly, the observed increase of ${D}_{Pt,\,Pd}^{SL/SM}$ with increasing ${C}_{Pt,\,Pd}^{SL}$ can be ascribed to the decrease of the$\,{\gamma }_{M{S}_{n/2}}^{SL\,}$ for Pt and Pd with increasing ${C}_{Pt,\,Pd}^{SL}$, or the increase of the ${\gamma }_{M{O}_{n/2}}^{SM\,}$for Pt and Pd with increasing ${C}_{Pt,\,Pd}^{SM}$. However, when ${C}_{Pt,\,Pd}^{SL}$ increases from 60 to 6000 ppm and from 26 to 2000 ppm, respectively, ${C}_{Pt,\,Pd}^{SM}$ increases only by a factor of ~2 (Fig. 2a, b), but ${D}_{Pt,\,Pd}^{SL/SM}$ increase by a factor of ~30–100 (Fig. 2c, d). Therefore, the ${\gamma }_{M{O}_{n/2}}^{SM\,}$for Pt and Pd may also be constant, and the increase of ${D}_{Pt,\,Pd}^{SL/SM}$ with increasing ${C}_{Pt,\,Pd}^{SL}$ most likely reflects that the$\,{\gamma }_{M{S}_{n/2}}^{SL\,}\,$for Pt and Pd decreases with increasing ${C}_{Pt,\,Pd}^{SL}$.

We take the advantage of available experimental results^16^ on coexisting Fe-Pt alloy and Fe-S-Pt sulfide liquid at 1200–1300 °C and 1 bar to thermodynamically understand the variation of the$\,{\gamma }_{M{S}_{n/2}}^{SL\,}\,$for Pt as a function of ${C}_{Pt}^{SL}$. Since the activity of Pt in the Fe-Pt alloy can be calculated thermodynamically^16^, we can calculate $\frac{1}{k}\cdot {\gamma }_{Pt{S}_{n/2}}^{SL}$ ($k$ is a constant as shown below) in the Fe-S-Pt sulfide liquid using the following approach. The partitioning of Pt between Fe-S-Pt sulfide liquid and Fe-Pt alloy can be written as:

6 $${{{{{\rm{Pt}}}}}}({{{{{\rm{alloy}}}}}})+n/4{{{{{{\rm{S}}}}}}}_{2}({{{{{\rm{gas}}}}}})={{{{{{\rm{PtS}}}}}}}_{{{{{{\rm{n}}}}}}/2}({{{{{\rm{sulfide}}}}}}\,{{{{{\rm{liquid}}}}}})$$

where *n* is the valence state of Pt in the Fe-S-Pt sulfide liquid. The equilibrium constant of Eq. (6) can be written as:

7 $${k}_{{{{{{\rm{Eq}}}}}}.(6)}=\frac{{\alpha }_{Pt{S}_{n/2}}^{SL}}{{\alpha }_{Pt}^{alloy}\cdot {(f{S}_{2})}^{n/4}}$$

and

8 $${k}_{{{{{{\rm{Eq}}}}}}.(6)}=\frac{{\gamma }_{Pt{S}_{n/2}}^{SL}\cdot {x}_{Pt{S}_{n/2}}^{SL}}{{\alpha }_{Pt}^{alloy}\cdot {(f{S}_{2})}^{n/4}}$$

which can be further arranged as:

9 $$\frac{1}{{k}_{{{{{{\rm{Eq}}}}}}.(6)}}\cdot {\gamma }_{Pt{S}_{n/2}}^{SL}=\frac{{\alpha }_{Pt}^{alloy}\cdot {(f{S}_{2})}^{n/4}}{{x}_{Pt{S}_{n/2}}^{SL}}$$

In Eqs. (7)–(9)$\,{\alpha }_{Pt}^{alloy}\,$and ${\alpha }_{Pt{S}_{n/2}}^{SL}$ are the activity of Pt and $Pt{S}_{n/2}$ in the Fe-Pt alloy and the Fe-S-Pt sulfide liquid, respectively;$\,{\gamma }_{Pt{S}_{n/2}}^{SL}$and ${x}_{Pt{S}_{n/2}}^{SL}$are the activity coefficient and mole fraction of $Pt{S}_{n/2}$ in the Fe-S-Pt sulfide liquid, respectively; and $f\,{S}_{2}$ is sulfur fugacity. Since Pt is present mainly as Pt^2+^ in sulfide liquid at geologically relevant redox conditions^16^, Eq. (9) can be written as:

10 $$\frac{1}{{k}_{{{{{{\rm{Eq}}}}}}.(6)}}\cdot {\gamma }_{PtS}^{SL}=\frac{{\alpha }_{Pt}^{alloy}\cdot {(f\,{S}_{2})}^{1/2}}{{x}_{PtS}^{SL}}$$

**Fig. 3:**
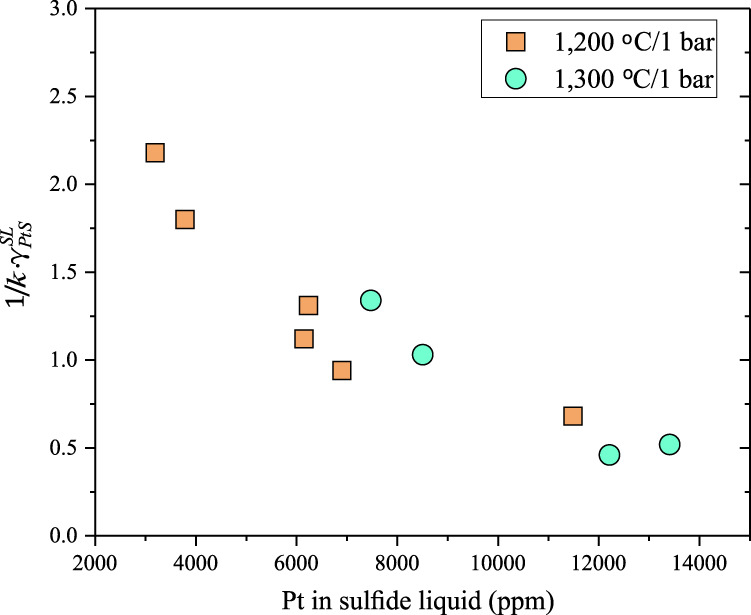
The calculated activity coefficient of PtS ($\frac{1}{k}\cdot {\gamma }_{PtS}^{SL}$) as a function of the Pt concentration in the Fe-S-Pt sulfide liquid. At the same temperature and pressure and similar *f*S_2_, $\frac{1}{k}\cdot {\gamma }_{PtS}^{SL}$ decreases with increasing the Pt concentration in the Fe-S-Pt sulfide liquid. Note that *k* is the equilibrium constant of Eq. (6) in the main text. The data used for these calculations were taken from Fonseca et al.^16^. Source data are provided in Supplementary Data 4.

Using the experimental results obtained at the same temperatures and similar $f{S}_{2}$ values in Fonseca et al.^16^, $\frac{1}{{k}_{{{{{{\rm{Eq}}}}}}.(6)}}\cdot {\gamma }_{PtS}^{SL}$ was calculated as a function of the Pt concentration in the Fe-S-Pt sulfide liquid (Supplementary Data 4). Figure 3 shows that $\frac{1}{{k}_{{{{{{\rm{Eq}}}}}}.(6)}}\cdot {\gamma }_{PtS}^{SL}$ decreases with increasing the Pt concentration in the Fe-S-Pt sulfide liquid. Therefore, ${\gamma }_{PtS}^{SL}$ must also decrease with increasing the Pt concentration in the Fe-S-Pt sulfide liquid at a given temperature, because ${k}_{{{{{{\rm{Eq}}}}}}.(6)}$ is a constant at a given temperature. These results thus support our inference that the$\,{\gamma }_{M{S}_{n/2}}^{SL\,}\,$for Pt and Pd decreases with increasing ${C}_{Pt,\,Pd}^{SL}$, and our explanations for the non-Henrian behavior of ${D}_{Pt,\,Pd}^{SL/SM}$. Here it should be noted that the dissolution of a fraction of Pt and/or Pd as sulfide species in the silicate melt^4,17^, which is not considered in Eqs. (1–5), does not change our explanations for the non-Henrian behavior of ${D}_{Pt,\,Pd}^{SL/SM}$.

Our experimental results and thermodynamic considerations conclusively demonstrate that ${D}_{Pt,\,Pd}^{SL/SM}$ follow a non-Henrian behavior. Therefore, the Pt and Pd concentration must be considered when ${D}_{Pt,\,Pd}^{SL/SM}$ are applied in a magmatic system, and the partitioning of Pt and Pd in the sulfide liquid is favored as the Pt and Pd concentration increases in the magma. The Pt and Pd concentration in sulfides of terrestrial mantle rocks and basalts is usually below 100 ppm^25–27^, so the ${D}_{Pt,\,Pd}^{SL/SM}$ values of < 10^4^, rather than the values of >10^5^ that have been widely accpeted^7,8,20^, should be used in terrestrial magmatic systems. We below show that the use of ${D}_{Pt,\,Pd}^{SL/SM}$ values of < 10^4^ well explains the observed Pt and Pd systematics of Earth’s mantle peridotites and MORB.

### Model the behavior of Pt and Pd during terrestrial magmatic process

**Fig. 4:**
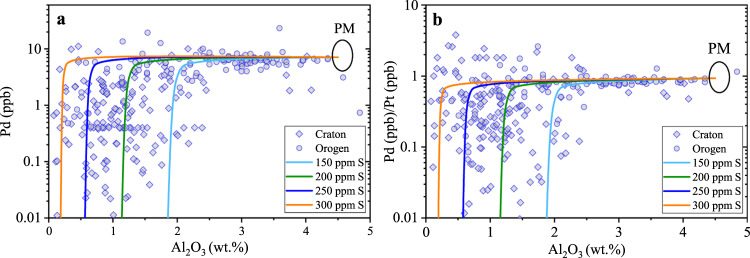
Comparison of the Pd content and Pd/Pt ratio of Earth’s mantle peridotites with those of our model calculations. **a, b** Our model calculations were performed by partial melting of Earth’s primitive mantle (PM), with Al_2_O_3_ content (wt.%) in the residual mantle as a proxy for the degree of partial melting. The used ${D}_{Pt,\,Pd}^{SL/SM}$ values are 4000 and 2000, respectively. The black ellipses refer to the Pd content and Pd/Pt ratio of the PM (refs. ^34, 35^). Mantle peridotite Pt, Pd, and Al_2_O_3_ data were taken from refs. ^28, 29^ and references therein. This figure illustrates that the Pd content and Pd/Pt ratio in Earth’s mantle peridotites can be largely explained by PM partial melting with ${D}_{Pt,\,Pd}^{SL/SM}$ values of 4000 and 2000, respectively. Source data are provided in Supplementary Data 5.

The newly determined ${D}_{Pt,\,Pd}^{SL/SM}$ as a function of ${C}_{Pt,\,Pd}^{SL}$ allow us to reconsider the behavior of Pt and Pd during partial melting of Earth’s mantle and MORB differentiation. A large dataset of PGE in Earth’s mantle peridotites has been established by previous studies^28,29^, and considerable variations of the PGE content in Earth’s mantle peridotites, as shown in Fig. 4, were explained as resulting from petrogenetic processes such as partial melting and the percolation of mantle fluids/melts^28,30^. To model the Pd content and Pt/Pd ratio in Earth’s mantle peridotites (Fig. 4 and Supplementary Data 5), a near-fractional melting model^31^ was used. Whole rock Al_2_O_3_ content was taken as a melt depletion indicator, and the melt extraction model parameters for Al_2_O_3_ were taken from ref. ^32^. During paritial melting, all sulfide was assumed to be sulfide liquid^33^. In addition, the following conditions were used: (i) the starting silicate composition is Earth’s primitive mantle^34,35^; (ii) the S concentration at sulfide saturation is 1000 ppm in the generated basaltic melt^36^; (iii) Earth’s primitive mantle contains 150–300 ppm S as sulfide (refs. ^37,38^); (σ) the ${D}_{Pt,\,Pd}^{SL/SM}$ during partial melting of Earth’s primitive mantle are 4000 and 2000, respectively; (τ) Pd is completely incompatible in silicate and oxide minerals^5^; (υ) the partition coefficients of Pt between mantle minerals and silicate melt are 0.009 for olivine, 0.8 for clinopyroxene, 2.2 for orthopyroxene, and 0.22 for spinel^39–42^. Figure 4 shows the modeled Pd content and Pd/Pt ratio in Earth’s mantle peridotites, which will cover the observed values, particularly if the variations of the S, Pt, and Pd content in Earth’s primitive mantle are considered. Accordingly, partial melting of Earth’s primitive mantle with ${D}_{Pt,\,Pd}^{SL/SM}$ values not exceeding 10^4^ can largely explain the observed Pt and Pd systematics of Earth’s mantle peridotites.

**Fig. 5:**
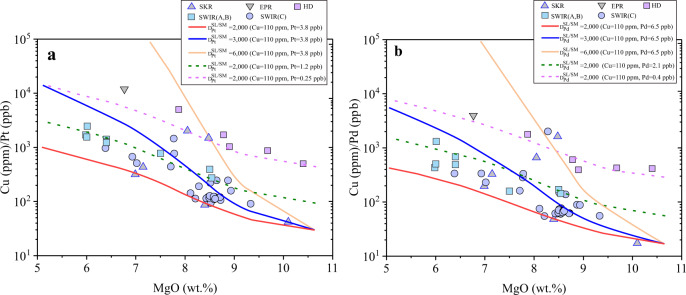
Modeled Cu/Pt and Cu/Pd ratios as a function of the MgO content during MORB differentiation. **a, b** The ${D}_{Pt,\,Pd}^{SL/SM}$ values of 2000–6000 were applied in the modeled differentiation trends, and the observed Cu/Pt and Cu/Pd ratios in MORB can be largely explained. Note that the solid and dashed differentiation lines were modeled using different initial Pt and Pd contents (see main text for details). The MORB Cu, Pt, and Pd data were taken from refs. ^5, 43^. SKR Southern Kolbeinsey Ridge, EPR East Pacific Rise; SWIR (A, B): Southwest Indian Ridge Zone A and B; SWIR(C): Southwest Indian Ridge Zone C, HD: HREE-depleted MORBs. Source data are provided in Supplementary Data 6.

We have also modeled the behavior of Pd and Pt during MORB differentiation (Fig. 5 and Supplementary Data 6). The Cu/Pt and Cu/Pd ratios are used as effective tools to identify the timing of sulfide liquid saturation during magmatic differentiation; the Cu/Pt and Cu/Pd ratios in magmas will increase if sulfide liquid saturation occurs^26,39,43^. To model the evolution trends of Cu/Pt and Cu/Pd during MORB differentiation, we used a fractional crystallization model and assumed that MORB crystallizes isobarically at 0.1 GPa. The relative mass fractions of crystallized minerals (olivine, clinopyroxene, and plagioclase), and the major element composition of the basaltic melt, during MORB differentiation were determined using the Petrolog3 program^44^. We used the SCSS model of Smythe et al.^45^ to calculate the S concentration at sulfide saturation in the basaltic melt and the mass of sulfide precipitated, with an assumption that S is removed as sulfide liquid which contains 46 wt.% Fe, 10 wt.% Ni, 7 wt.% Cu, 2 wt.% O, and 35 wt.% S (ref. ^26^). Three different groups of Cu, Pt, and Pd content (Cu = 110 ppm, Pt = 3.8 ppb, and Pd = 6.5 ppb; Cu = 110 ppm, Pt = 1.2 ppb, and Pd = 2.1 ppb; Cu = 110 ppm, Pt = 0.25 ppb, and Pd = 0.40 ppb) were used for the parental MORB to differentiate, considering that the parental MORB could have very different Pt and Pd contents^5,43^. The used ${D}_{Pt,\,Pd}^{SL/SM}$ values ranged from 2000 to 6000, and the partition coefficients of Pt between minerals and silicate melt are 0.009 for olivine, 0.8 for clinopyroxene, and 0.3 for plagioclase^39,46^. The partition coefficients of Cu between sulfide liquid and basaltic melt were fixed at 800 (refs. ^23,24,47^). The partition coefficients of Cu between minerals and basaltic melt are 0.05 for olivine, 0.049 for clinopyroxene, and 0.115 for plagioclase^48,49^. Figure 5 shows that during MORB differentiation, the Cu/Pt and Cu/Pd ratios increase with decreasing the MgO content (wt.%) in the silicate melt, indicating the saturation of sulfide liquid during MORB differentiation^26,50,51^. Figure 5 also shows that the modeled Cu/Pt and Cu/Pd ratios, using ${D}_{Pt,\,Pd}^{SL/SM}$ values of 2000–6000, well cover the observed Cu/Pt and Cu/Pd ratios in the differentiated MORB. Therefore, the observed Pt and Pd systematics of MORB can be explained by the use of ${D}_{Pt,\,Pd}^{SL/SM}$ values of 2000–6000 for MORB differentiation.

### Implications for the geochemical behavior of PGE in planetary magmas

The modeling results shown in Figs. 4 and 5 demonstrate that the use of ${D}_{Pt,\,Pd}^{SL/SM}$ values of 2000–6000 well explains the observed Pd and Pt systematics of Earth’s mantle peridotites and MORB, with ${D}_{Pt,\,Pd}^{SL/SM}$ values of >10^5^ not required. The use of ${D}_{PGE}^{SL/SM}$ values of >10^5^ readily explains the extreme enrichments of PGE in a volumetrically small sulfide fraction and the formation of world-class PGE deposits, such as the Stillwater and Bushveld Complex-hosted reef deposits^6,7,52^. However, our findings, based on both experimental results (Fig. 2) and modeling results (Figs. 4 and 5), suggest that the application of ${D}_{Pt,\,Pd}^{SL/SM}$ values of >10^5^ in terrestrial magmas is inappropriate, and sulfide immiscibility alone cannot explain the observed high Pt and Pd abundance in the Stillwater and Bushveld Complex-hosted reef deposits, which have PGE enrichment factors of 10^5^–10^6^ (refs. ^6,7,19^). The other mechanisms, such as direct crystallization of PGE-bearing minerals from magmas^53,54^, and/or secondary enrichment of PGE in immiscible sulfide liquid^55^, may be necessary conditions for the formation of world-class magmatic PGE sulfide deposits.

Our experimental and modeling results also have significant implications for planetary accretion. The ${D}_{Pt,\,Pd}^{SL/SM}$ values of < 10^4^ when ${C}_{Pt,\,Pd}^{SL}$ < 100 ppm suggest that the capacity of segregating sulfide liquid (Hadean matte) in extracting Pt and Pd from Earth’s solidifying magma ocean is not as strong as previously thought^3,56^. In this context, the models proposed for Earth’s accretion and core-formation based on ${D}_{Pt,\,Pd}^{SL/SM}$ values of >10^5^ (refs. ^3,56^) need to be revised to explain the depleted but near-chondritic relative PGE abundance in the bulk silicate Earth. The non-Henrian behavior of ${D}_{Pt,\,Pd}^{SL/SM}$ may also suggest that the partitioning of PGE between metallic and silicate melts does not obey Henry’s law either. A dependence of the partition coefficients of Pd between metallic and silicate melts (160–3 × 10^5^) on the Pd concentration in the metallic melt (3000–89,000 ppm) has indeed been observed by Wheeler et al.^57^. Consequently, the distribution of PGE between planetary core and mantle may be controlled by not only temperature and pressure^1–3^ but also the PGE concentration in the system. We speculate that it may be possible that the depleted but near-chondritic relative PGE abundance in planetary silicate mantles^58^ can be explained by core-formation alone, i.e., a late veneer not being required, if all PGE follow a non-Henrian partitioning behavior between metallic and silicate melts. This speculation deserves future investigations.

Our study conclusively demonstrates that the partitioning of PGE between sulfide liquid and silicate melt is more complex than previously thought. Future laboratory studies must be performed to fully understand the partitioning behavior of PGE between metallic melt, sulfide liquid, and silicate melt, with PGE concentrations close to the natural systems. This will be critically important for tracing many planetary processes, such as the formation of magmatic PGE sulfide deposits in Earth’s crust, the origin of the near-chondritic relative PGE abundance in planetary silicate mantles, and the accretion and differentiation of terrestrial planets.

## Methods

### Starting materials

Starting materials loaded in each sample capsule included ~60–70 wt.% of a synthetic silicate and ~30–40 wt.% sulfide (FeS). The synthetic silicate with major element compositions similar to those of average MORB were prepared from analytical grade oxides and carbonates. To minimize absorbed water, SiO_2_, TiO_2_, Al_2_O_3_, Cr_2_O_3_ and MgO powder were each heated overnight at 1000 °C, MnO_2_ at 400 °C, CaCO_3_ at 200 °C, and Na_2_CO_3_ and K_2_CO_3_ at 110 °C. After drying, all the oxides and carbonates were mixed and ground in acetone, then dried at room temperature overnight. Well-mixed oxide and carbonate powder was sintered in a high purity alumina crucible at 1000 °C overnight to decarbonate. The decarbonated powder was finally mixed with FeO powder and ground in acetone, so as to limit the introduction of ferric iron into subsequent partitioning experiment, which was proved to be an efficient way to prevent the formation of PGE nuggets in silicate melt^21^. In the forward experiments, ~20–20,000 ppm Pt or Pd was doped in the sulfide powder, which was then sintered in a graphite capsule at 1100 °C and 1.5 GPa for 10 h. In the reversal experiments, ~200–7000 ppm Pd or 1000 ppm Pt was physically mixed with the silicate powder in acetone in an agate mortar, which was then dried at room temperature. All dried silicate and sulfide materials were stored in a vacuumed oven at 110 °C for > 24 h before loading into graphite capsules for high-pressure experiments.

### High temperature and pressure experiments

All experiments were conducted at 1.0 GPa and 1400 °C in an end-loaded solid media piston cylinder apparatus, using 3/4-inch diameter talc-Pyrex assemblies with graphite heaters. Pressure was calibrated against the quartz-coesite and kyanite-sillimanite transitions, and a friction correction of 18% was applied to the nominal pressure. The total pressure uncertainty is less than 0.1 GPa. The experimental temperatures were monitored by C-type (W_95_Re_05_-W_74_Re_26_) thermocouples, and temperature was controlled to ±2 °C and was accurate to ±10 °C. For each experiment, in order to reduce the porosity of the graphite capsules and thus prevent leakage of sulfide liquid, the sample was first heated to 850 °C and held for 2–4 h and then raised to the target temperature. The experimental durations ranged from 24 to 96 h. After quenching by turning off electric power to the graphite heaters, the recovered capsules were sectioned longitudinally into two halves, mounted in epoxy, and polished for EPMA and LA–ICP–MS analyses.

### Electron microprobe analyses of major elements

Major elements compositions of the quenchend sulfide liquid and silicate melt were measured with a JEOL JXA-8230 microprobe. The analyses were performed in wavelength-dispersive mode, and a PAP matrix correction was applied to the raw data. For the measurements of major elements in the silicate melt, a defocused beam of 20 µm, 15 kV accelerating voltage, and 10 nA beam current were used for both the standardizations and sample measurements. Natural and synthetic standards were used to calibrate the instrument, and the used standards were andradite (Si), MnTiO_3_ (Ti), spinel (Al), metal Fe (Fe), MnTiO_3_ (Mn), forsterite (Mg), wollastonite (Ca), albite (Na), orthoclase (K), Gallium phosphite (P), and metal Ni (Ni). The peak counting time was 20 s except for Na and K, which were measured for 10 s. The quenched sulfide liquid was analyzed with 20 kV acceleration voltage and 20 nA beam current. Fe and S were calibrated on a synthetic pyrrhotite with a well-known Fe:S ratio, Ni, Co, Cu, Pd and Pt were calibrated on pure metals, and O was calibrated on magnetite. A defocused beam of 30 µm diameter was used for all standardizations and sample measurements, as this was sufficient to average small-scale quench-phase inhomogeneity. Sulfur in the quenched silicate melts was analyzed with 50 nA beam current and 60 s peak counting time using the synthetic pyrrhotite standard.

### LA–ICP–MS analyses of Pt and Pd

Major and trace elements of the quenched silicate melt and sulfide liquid were measured using a laser–ablation ICP–MS. These analyses were carried out on an Agilent 7900 Quadrupole ICP-MS coupled to a Photon Machines Analyte HE 193-nm ArF Excimer Laser Ablation system. The quenched silicate melt was analyzed with 10 Hz, 80 mJ, and laser beam sizes of 90–110 μm, whereas the quenched sulfide liquid was analyzed with 7 Hz, 70 mJ, and laser beam sizes of 50–60 μm. A typical time-resolved analysis involved ~20 s of background acquisition, followed by laser ablation for 40 s. The sample chamber was flushed with He at a rate of 0.4 L/min, to which 5 ml/min H_2_ was added on the way to the ICP–MS. The isotopes ^23^Na, ^24^Mg, ^27^Al, ^29^Si, ^32^P, ^39^K, ^42^Ca, ^49^Ti, ^52^Cr, ^55^Mn, ^57^Fe, ^59^Co, ^62^Ni, ^65^Cu, ^75^As, ^121^Sb, and ^209^Bi were measured with a dwell time of 10 ms. To precisely measure ^105^Pd and ^195^Pt in the silicate melt, ^105^Pd and ^195^Pt were measured with a dwell time of 50 ms. NIST SRM 610 glass was used as external standard for all analyses, which contains 3.15 ppm Pt and 1.05 ppm Pd^59^, whereas Si and Fe determined by electron microprobe were used as the internal standard for the silicate melt and sulfide, respectively. Mass-1 sulfide standard was used to check the accuracy of the LA-ICP-MS analyses. Repeated analyses of the Mass-1 sulfide standard after analyzing each ten sample spots yielded Pt and Pd concentrations consistent with the certified values within 6–17% relative, which demonstrates the validity of using NIST SRM 610 glass as the standard for measuring the Pt and Pd concentration in the quenched sulfide liquid.

As done in previous studies^60,61^, the detection limits (DL) for measuring the Pt and Pd concentration (${C}_{Pt,\,Pd}^{SM}$) in the silicate melt were calculated as three times the standard deviation of three replicate measurements of a sample that contains zero analyte^62^. Each analytical point has a own DL because the laser beam size and the interval of signal used for integration may change, althouth a dwell time of 50 ms was always used. The calculated minimum to maximum DL_min-max_ values for the analyses of each sample were given in Supplementary Data 1, which are 3 (min)–7 ppb (max) for Pt and 3 (min)–8 ppb (max) for Pd. Our measured ${C}_{Pt,\,Pd}^{SM}$ are significantly higher than DL_min-max_. Most samples have ${C}_{Pt,\,Pd}^{SM}$/DL_ave_ ratios in the range of 3–39 (DL_ave_ = average of DL values for a given sample; Supplementary Data 1). Only five of twenty-two samples show relatively low ${C}_{Pt,\,Pd}^{SM}$/ DL_ave_ ratios (2.6–2.9); however, these ${C}_{Pt,\,Pd}^{SM}$ values are still well around the quantification limits. The low ${C}_{Pt,\,Pd}^{SM}$ (down to ~4 ppb) determined by previous study was confirmed to be reliable under similar analytical conditions^4,21^. In addition, the measured ${C}_{Pt,\,Pd}^{SM}$ values of most of our samples have one sigma standard deviation (σ) < 30% relative (Supplementary Data 1). All of these demonstrate the high sensitivity of our LA–ICP–MS analyses of Pt and Pd in the silicate melt and the homogeneous distribition of Pt and Pd in the silicate melt.

## Supplementary information

Supplementary Information

Peer Review File

Description of Additional Supplementary Files

Supplementary Data 1

Supplementary Data 2

Supplementary Data 3

Supplementary Data 4

Supplementary Data 5

Supplementary Data 6

## Data Availability

All data supporting the findings of this study are available within the paper and supplementary information and data files (Supplementary Data 1–6). Additional data related to this paper may be requested from the authors.

## References

[1] Righter K, Humayun M, Danielson L (2008). Partitioning of palladium at high pressures and temperatures during core formation. Nat. Geosci..

[2] Mann U, Frost DJ, Rubie DC, Becker H, Audétat A (2012). Partitioning of Ru, Rh, Pd, Re, Ir and Pt between liquid metal and silicate at high pressures and high temperatures - Implications for the origin of highly siderophile element concentrations in the Earth’s mantle. Geochim. Cosmochim. Acta.

[3] Rubie DC (2016). Highly siderophile elements were stripped from Earth’s mantle by iron sulfide segregation. Science.

[4] Mungall JE, Brenan JM (2014). Partitioning of platinum-group elements and Au between sulfide liquid and basalt and the origins of mantle-crust fractionation of the chalcophile elements. Geochim. et. Cosmochim. Acta.

[5] Rehkämper M (1999). Ir, Ru, Pt, and Pd in basalts and komatiites: new constraints for the geochemical behavior of the platinum-group elements in the mantle. Geochim. Cosmochim. Acta.

[6] Mungall JE, Naldrett AJ (2008). Ore Deposits of the Platinum-Group Elements. Elements.

[7] Barnes S-J, Ripley EM (2015). Highly Siderophile and Strongly Chalcophile Elements in Magmatic Ore Deposits. Rev. Mineral. Geochem..

[8] Brenan JM, Bennett NR, Zajacz Z (2015). Experimental Results on Fractionation of the Highly Siderophile Elements (HSE) at Variable Pressures and Temperatures during Planetary and Magmatic Differentiation. Rev. Mineral. Geochem..

[9] Stone W, Crocket J (1990). & Fleet Partitioning of palladium, iridium, platinum, and gold between sulfide liquid and basalt melt at 1200 °C. Geochim. Cosmochim. Acta.

[10] Fleet M, Crocket J, Stone WJ (1996). Partitioning of platinum-group elements (Os, Ir, Ru, Pt, Pd) and gold between sulfide liquid and basalt melt. Geochim. Cosmochim. Acta.

[11] Crocket J, Fleet M (1997). Implications of composition for experimental partitioning of platinum-group elements and gold between sulfide liquid and basalt melt: the significance of nickel content. Geochim. Cosmochim. Acta.

[12] Fleet M, Crocket J, Liu M, Stone W (1999). Laboratory partitioning of platinum-group elements (PGE) and gold with application to magmatic sulfide–PGE deposits. Lithos.

[13] Bezmen N, Asif M, Brügmann G, Romanenko I, Naldrett A (1994). Distribution of Pd, Rh, Ru, Jr, Os, and Au between sulfide and silicate metals. Geochim. Cosmochim. Acta.

[14] Andrews DR, Brenan JMJ (2002). The solubility of ruthenium in sulfide liquid: implications for platinum group mineral stability and sulfide melt–silicate melt partitioning. Chem. Geol..

[15] Fonseca ROC, Mallmann G, O’Neill HSC, Campbell IH, Laurenz V (2011). Solubility of Os and Ir in sulfide melt: Implications for Re/Os fractionation during mantle melting. Earth Planet. Sci. Lett..

[16] Fonseca ROC, Campbell IH, O’Neill HSC, Allen CM (2009). Solubility of Pt in sulphide mattes: Implications for the genesis of PGE-rich horizons in layered intrusions. Geochim. Cosmochim. Acta.

[17] Laurenz V (2013). The solubility of palladium and ruthenium in picritic melts: 2. Eff. Sulfur. Geochim. Cosmochim. Acta.

[18] Brenan JM (2008). Re–Os fractionation by sulfide melt–silicate melt partitioning: a new spin. Chem. Geol..

[19] Barnes SJ, Holwell DA, Le Vaillant M (2017). Magmatic Sulfide Ore Deposits. Elements.

[20] Day JMD (2018). Geochemical constraints on residual metal and sulfide in the sources of lunar mare basalts. Am. Mineral..

[21] Médard E, Schmidt MW, Wälle M, Keller NS, Günther D (2015). Platinum partitioning between metal and silicate melts: Core formation, late veneer and the nanonuggets issue. Geochim. Cosmochim. Acta.

[22] Médard E, McCammon CA, Barr JA, Grove TL (2008). Oxygen fugacity, temperature reproducibility, and H_2_O contents of nominally anhydrous piston-cylinder experiments using graphite capsules. Am. Mineralogist.

[23] Li Y, Audétat A (2012). Partitioning of V, Mn, Co, Ni, Cu, Zn, As, Mo, Ag, Sn, Sb, W, Au, Pb, and Bi between sulfide phases and hydrous basanite melt at upper mantle conditions. Earth Planet. Sci. Lett..

[24] Kiseeva ES, Wood BJ (2013). A simple model for chalcophile element partitioning between sulphide and silicate liquids with geochemical applications. Earth Planet. Sci. Lett..

[25] Alard O, Griffin WL, Lorand JP, Jackson SE, O’Reilly SYJN (2000). Non-chondritic distribution of the highly siderophile elements in mantle sulphides. Nature.

[26] Patten C, Barnes S-J, Mathez EA, Jenner FE (2013). Partition coefficients of chalcophile elements between sulfide and silicate melts and the early crystallization history of sulfide liquid: LA-ICP-MS analysis of MORB sulfide droplets. Chem. Geol..

[27] Peach C, Mathez E, Keays RJ (1990). Sulfide melt-silicate melt distribution coefficients for noble metals and other chalcophile elements as deduced from MORB: Implications for partial melting. Geochim. Cosmochim. Acta.

[28] Lorand J-P, Luguet A, Alard O (2013). Platinum-group element systematics and petrogenetic processing of the continental upper mantle: a review. Lithos.

[29] Barnes SJ, Mungall JE, Maier WD (2015). Platinum group elements in mantle melts and mantle samples. Lithos.

[30] Lorand J-P, Luguet A (2015). Chalcophile and Siderophile Elements in Mantle Rocks: trace Elements Controlled By Trace Minerals. Rev. Mineral. Geochem..

[31] Lee C-TA (2012). Copper systematics in arc magmas and implications for crust-mantle differentiation. Science.

[32] Walter MJJ (2003). Melt extraction and compositional variability in mantle lithosphere. TrGeo.

[33] Zhang Z, Hirschmann MM (2016). Experimental constraints on mantle sulfide melting up to 8 GPa. Am. Mineralogist.

[34] Becker H (2006). Highly siderophile element composition of the Earth’s primitive upper mantle: Constraints from new data on peridotite massifs and xenoliths. Geochim. Cosmochim. Acta.

[35] Fischer-Gödde M, Becker H, Wombacher F (2011). Rhodium, gold and other highly siderophile elements in orogenic peridotites and peridotite xenoliths. Chem. Geol..

[36] Mavrogenes JA, O’Neill HSC (1999). The relative effects of pressure, temperature and oxygen fugacity on the solubility of sulfide in mafic magmas. Geochim. Cosmochim. Acta.

[37] Salters VJ, Stracke A (2004). Composition of the depleted mantle. Geochem. Geophys. Geosyst..

[38] McDonough WF, Sun S-S (1995). The composition of the Earth. Chem. Geol..

[39] Park J-W, Campbell IH, Arculus RJ (2013). Platinum-alloy and sulfur saturation in an arc-related basalt to rhyolite suite: evidence from the Pual Ridge lavas, the Eastern Manus Basin. Geochim. Cosmochim. Acta.

[40] Brenan JM, McDonough WF, Dalpé C (2003). Experimental constraints on the partitioning of rhenium and some platinum-group elements between olivine and silicate melt. Earth Planet. Sci. Lett..

[41] Brenan JM, Finnigan CF, McDonough WF, Homolova V (2012). Experimental constraints on the partitioning of Ru, Rh, Ir, Pt and Pd between chromite and silicate melt: The importance of ferric iron. Chem. Geol..

[42] Ely JC, Neal CR (2003). Using platinum-group elements to investigate the origin of the Ontong Java Plateau, SW Pacific. Chem. Geol..

[43] Yang AY (2014). Chalcophile elemental compositions of MORBs from the ultraslow-spreading Southwest Indian Ridge and controls of lithospheric structure on S-saturated differentiation. Chem. Geol..

[44] Danyushevsky, L. V. & Plechov, P. Petrolog3: Integrated software for modeling crystallization processes. *Geochem. Geophys. Geosyst.* **12**, 7 (2011).

[45] Smythe DJ, Wood BJ, Kiseeva ES (2017). The S content of silicate melts at sulfide saturation: new experiments and a model incorporating the effects of sulfide composition. Am. Mineral..

[46] Chazey WJ, Neal CR (2005). Platinum-group element constraints on source composition and magma evolution of the Kerguelen Plateau using basalts from ODP Leg 183. Geochim. Cosmochim. Acta.

[47] Kiseeva ES, Wood BJ (2015). The effects of composition and temperature on chalcophile and lithophile element partitioning into magmatic sulphides. Earth Planet. Sci. Lett..

[48] Liu X, Xiong X, Audétat A, Li Y (2015). Partitioning of Cu between mafic minerals, Fe–Ti oxides and intermediate to felsic melts. Geochim. Cosmochim. Acta.

[49] Liu X (2014). Partitioning of copper between olivine, orthopyroxene, clinopyroxene, spinel, garnet and silicate melts at upper mantle conditions. Geochim. Cosmochim. Acta.

[50] Jenner FE, O’Neill HSC, Arculus RJ, Mavrogenes JA (2010). The Magnetite Crisis in the Evolution of Arc-related Magmas and the Initial Concentration of Au, Ag and Cu. J. Pet..

[51] Li Y (2014). Comparative geochemistry of rhenium in oxidized arc magmas and MORB and rhenium partitioning during magmatic differentiation. Chem. Geol..

[52] Mudd GM (2012). Key trends in the resource sustainability of platinum group elements. Ore Geol. Rev..

[53] Kamenetsky VS, Zelenski MJG (2020). Origin of noble-metal nuggets in sulfide-saturated arc magmas: a case study of olivine-hosted sulfide melt inclusions from the Tolbachik volcano (Kamchatka, Russia). Geology.

[54] Anenburg M, Mavrogenes JA (2016). Experimental observations on noble metal nanonuggets and Fe-Ti oxides, and the transport of platinum group elements in silicate melts. Geochim. Cosmochim. Acta.

[55] Anenburg M, Mavrogenes JA (2020). Noble metal nanonugget insolubility in geological sulfide liquids. Geology.

[56] Laurenz V, Rubie DC, Frost DJ, Vogel AK (2016). The importance of sulfur for the behavior of highly-siderophile elements during Earth’s differentiation. Geochim. Cosmochim. Acta.

[57] Wheeler KT, Walker D, McDonough WF (2011). Pd and Ag metal-silicate partitioning applied to Earth differentiation and core-mantle exchange. Meteorit. Planet. Sci..

[58] Dale CW (2012). Late accretion on the earliest planetesimals revealed by the highly siderophile elements. Science.

[59] Sylvester PJ, Eggins SMJ (1997). Analysis of Re, Au, Pd, Pt and Rh in NIST glass certified reference materials and natural basalt glasses by laser ablation ICP‐MS. Geostand. Newsl..

[60] Jégo S, Pichavant M, Mavrogenes JA (2010). Controls on gold solubility in arc magmas: an experimental study at 1000 °C and 4 kbar. Geochimica et. Cosmochimica Acta.

[61] Jégo S, Pichavant M (2012). Gold solubility in arc magmas: experimental determination of the effect of sulfur at 1000 °C and 0.4 GPa. Geochim. Cosmochim. Acta.

[62] Longerich HP, Jackson SE, Günther D (1996). Inter-laboratory note. Laser ablation inductively coupled plasma mass spectrometric transient signal data acquisition and analyte concentration calculation. J. Anal. At. Spectrom..

