# Supplementary material for: Breaking of Henry’s law for sulfide liquid–basaltic melt partitioning of Pt and Pd
Source: Nat Commun. 2021 Oct 13;12:5994. doi: 10.1038/s41467-021-26311-x (PMC8514440; doi:10.1038/s41467-021-26311-x)
Supplement: Supplementary file 1 — Supplementary Information [file 41467_2021_26311_MOESM1_ESM.pdf]

Supplementary Information for

Breaking of Henry's law for sulfide liquid–basaltic melt partitioning of Pt and Pd

Mingdong Zhang[1, 2, 3], Yuan Li[1, 2*]

[1]State Key Laboratory of Isotope Geochemistry, Guangzhou Institute of Geochemistry, Chinese Academy of Sciences, Guangzhou 510640, China.

[2]CAS Center for Excellence in Deep Earth Science, Guangzhou, 510640, China.

[3]College of Earth and Planetary Sciences, University of the Chinese Academy of Sciences, Beijing 100049, China.

The Supplementary Information includes Supplementary Figs. 1-5.

* Corresponding author. Email address: Yuan.Li@gig.ac.cn

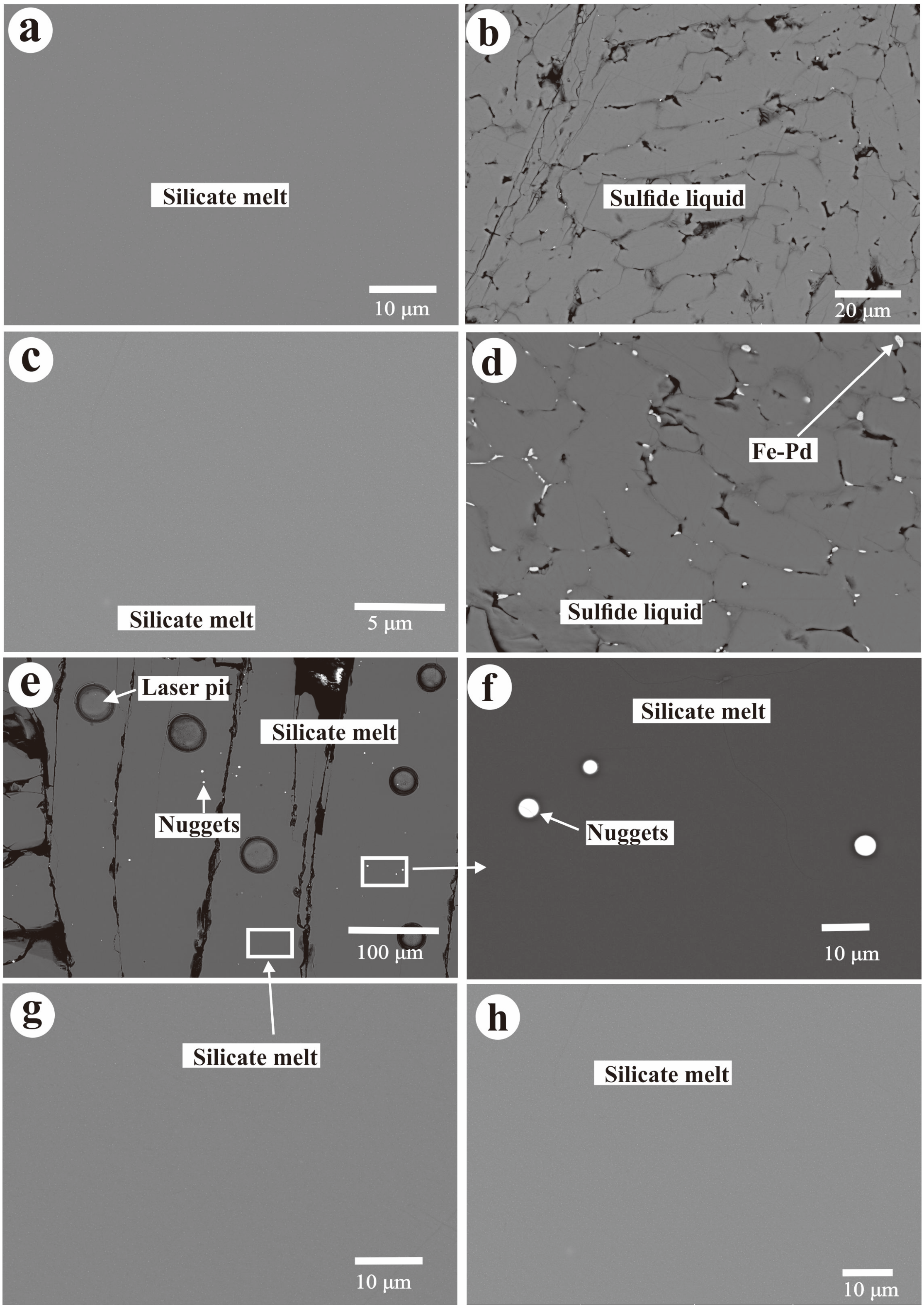

Supplementary Figure 1. **Selected back-scattered electron images showing characteristic features of the run products of sulfide liquid – silicate melt partitioning experiments.** (**a**) The quenched silicate melt produced in run Z15, showing homogeneously distributed small sulfide dots (<0.1 μm) produced during quench. (**b**) Detailed texture of the quenched sulfide liquid with 153 ppm Pt in run Z15.

(**c**) The quenched silicate melt produced in run Z26. (**d**) The quenched sulfide liquid with 5,600 ppm Pd in run Z26, which includes large pyrrhotite crystals and exsolved Fe-Pd alloys. (**e**) One reversal experiment (run Z33) for Pt partitioning, showing small amounts of sulfide nuggets (1-5 μm) in the silicate melt. (**f**) Closer view of the quenched silicate melt with sulfide nuggets in (**e**). (**g**) Closer view of the quenched silicate melt in (**e**), which is free of sulfide nuggets. (**h**) The quenched silicate melt produced in run Z27, which is free of sulfide nuggets.

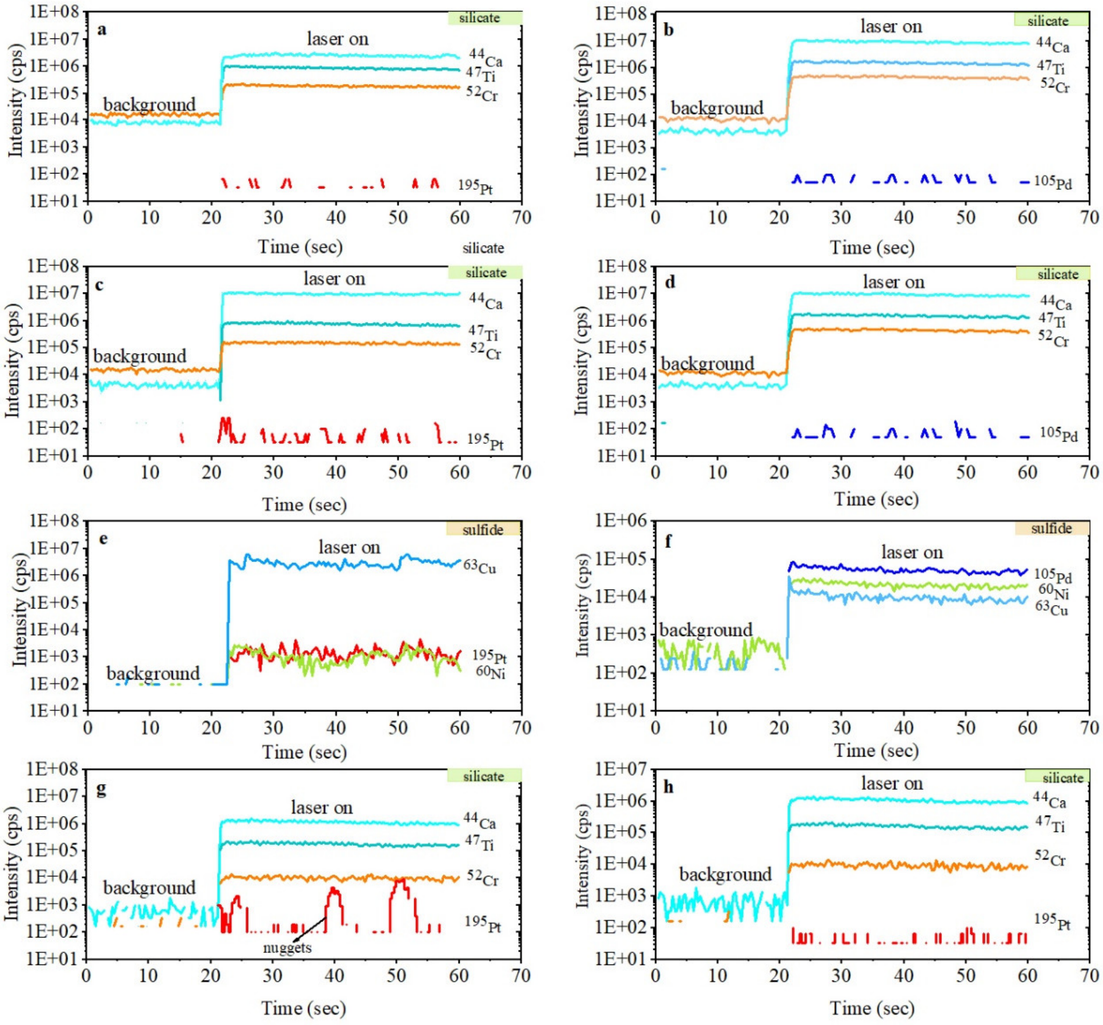

Supplementary Figure 2. **Time-resolved LA-ICP-MS signals of Pt and Pd and other elements in the quenched silicate melt and sulfide liquid**. The signals of Pt and Pd in both silicate melt and sulfide liquid are constant (**a**: run Z14; **b**: run Z22; **c**: run Z16; **d**: run Z27; e: run Z15; **f**: run Z26; **h**: run Z33). In (**g**), a typical ^{195}Pt signal of sulfide nuggets in the silicate melt, taken from one reversal experiment for Pt partitioning (run Z33).

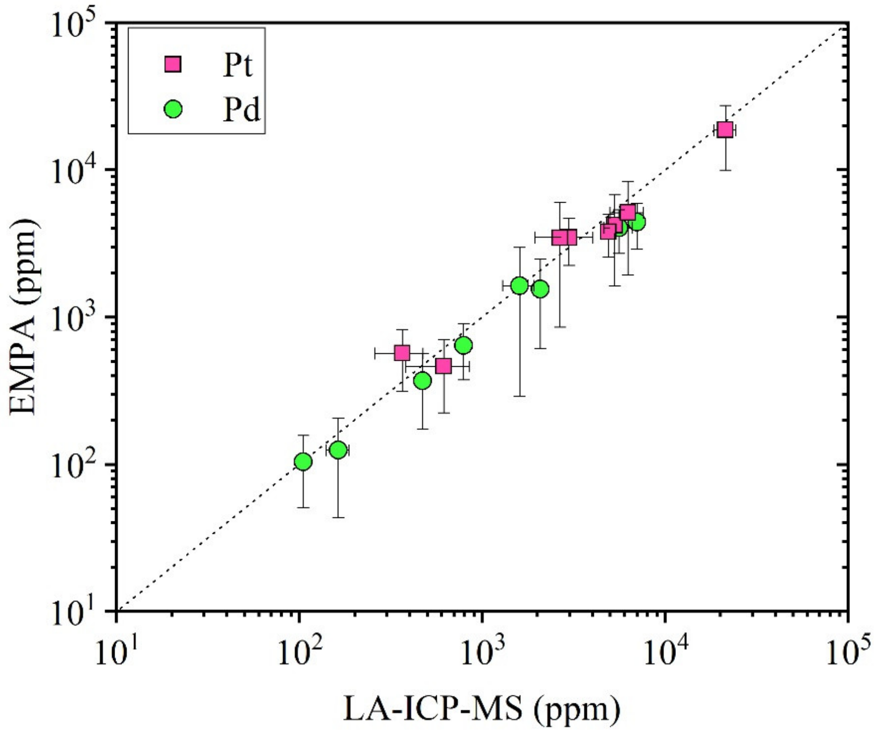

Supplementary Figure 3. **Comparison of the sulfide liquid Pt and Pd contents measured by LA-ICP-MS and electron probe micro-analyzer (EPMA).** This figure shows that EPMA and LA-ICP-MS yielded identical values within errors when the Pt and Pd concentrations in the sulfide liquid are above 100 ppm (data from Supplementary Data 3).

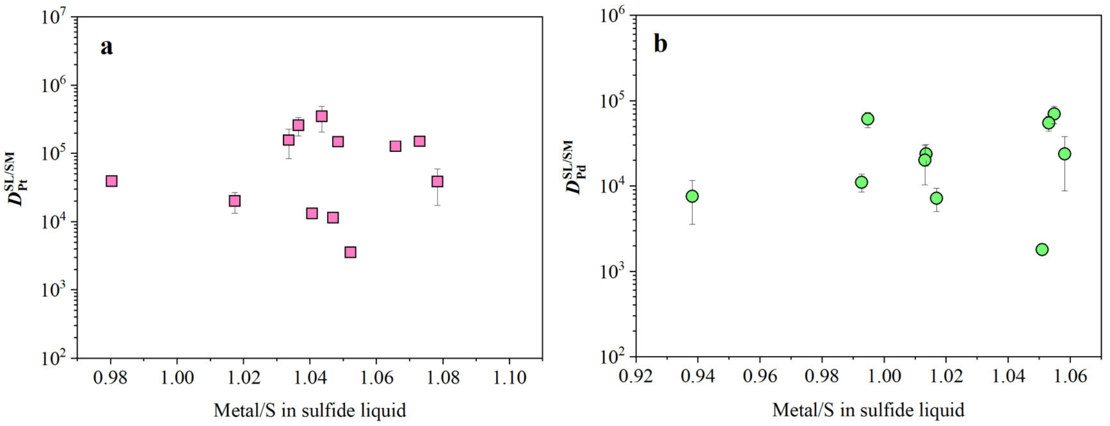

Supplementary Figure 4. $\boldsymbol{D_{Pt,Pd}^{SL/SM}}$ **as a function of the atomic metal/S ratio in sulfide liquid.** Note that $D_{Pt,Pd}^{SL/SM}$ are not considerably affected by the atomic metal/S ratio in sulfide liquid.

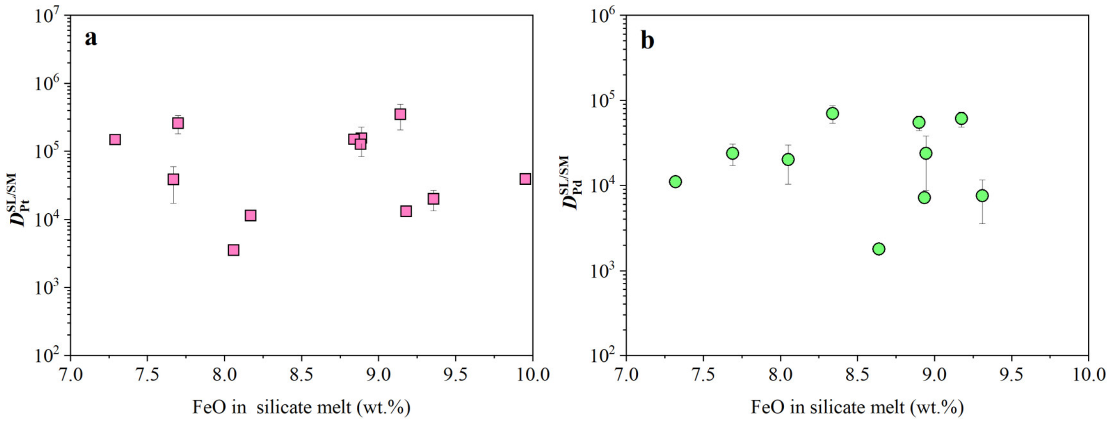

Supplementary Figure 5. $\boldsymbol{D_{Pt,Pd}^{SL/SM}}$ **as a function of the FeO content in the silicate melt.** Note that $D_{Pt,Pd}^{SL/SM}$ are not considerably affected by the FeO content in the silicate melt.
