# Supplementary material for: Breaking of Henry’s law for sulfide liquid–basaltic melt partitioning of Pt and Pd
Source: Nat Commun. 2021 Oct 13;12:5994. doi: 10.1038/s41467-021-26311-x (PMC8514440; doi:10.1038/s41467-021-26311-x)
Supplement: Supplementary file 3 — Description of Additional Supplementary Files [file 41467_2021_26311_MOESM3_ESM.pdf]

Description of Additional Supplementary Files

File name: Supplementary Data 1
Description: Summary of the experimental conditions and products and the calculated partition coefficients for Pt and Pd.

File name: Supplementary Data 2
Description: Major element compositions of the quenched silicate melts determined by EPMA (wt.%).

File name: Supplementary Data 3
Description: Major and trace element compositions of the quenched sulfide liquid determined by EPMA and LA-ICP-MS.

File name: Supplementary Data 4
Description: The calculated activity coefficient ($1/k \cdot \gamma_{\mathrm{PtS}}^{SL}$) of PtS in the Fe-S-Pt sulfide liquid.

File name: Supplementary Data 5
Description: The modeled variation of Pt and Pd in mantle peridotites as a function of Al_2O_3 during partial melting of Earth's primitive mantle.

File name: Supplementary Data 6
Description: The modeled variation of Cu, Pt, and Pd during MORB differentiation.
